# Barriers and Facilitators to Implementing a Digital Adherence Technology for Tuberculosis Treatment Supervision in Uganda: Qualitative Study

**DOI:** 10.2196/38828

**Published:** 2023-05-30

**Authors:** Anna Leddy, Joseph Ggita, Christopher A Berger, Alex Kityamuwesi, Agnes Nakate Sanyu, Lynn Kunihira Tinka, Rebecca Crowder, Stavia Turyahabwe, Achilles Katamba, Adithya Cattamanchi

**Affiliations:** 1 Center for Tuberculosis and Division of Pulmonary and Critical Care Medicine University of California, San Francisco San Francisco, CA United States; 2 Uganda Tuberculosis Implementation Research Consortium Kampala Uganda; 3 National Tuberculosis and Leprosy Program Uganda Ministry of Health Kampala Uganda

**Keywords:** digital adherence technology, gender norms, tuberculosis, adherence, sub-Saharan Africa

## Abstract

**Background:**

Ensuring the completion of treatment for tuberculosis (TB) remains a key challenge in many high-burden countries. 99DOTS is a low-cost digital adherence technology that has emerged as a promising tool for monitoring and supporting TB treatment completion.

**Objective:**

We aimed to understand the feasibility and acceptability of 99DOTS, a mobile phone–based TB treatment support method, and characterize barriers and facilitators to its implementation during a pragmatic trial in Uganda.

**Methods:**

Between April 1 and August 31, 2021, we conducted in-depth interviews with people with TB and key informant interviews with health workers and district and regional TB officers involved in the implementation of 99DOTS at 18 health facilities in Uganda. Semistructured interview guides were informed by the capability, opportunity, motivation, and behavior (COM-B) model and explored perceptions of, and experiences with, 99DOTS, including barriers and facilitators to its use. Qualitative analysis was conducted using the framework approach.

**Results:**

Interviews were conducted with 30 people with TB, 12 health workers, and 7 TB officers. All people with TB, health workers, and TB officers noted that 99DOTS supported and encouraged people with TB to take their anti-TB medication, facilitated treatment monitoring, and improved relationships between people with TB and health workers. Participants also liked that the platform was free, easy to use, and improved TB treatment outcomes. Barriers to 99DOTS implementation for some people with TB were related to limited literacy, including technology literacy; limited access to electricity to charge their mobile phone to make dosing confirmation calls; and poor network connection. Gender differences in 99DOTS uptake also emerged. Specifically, women with TB were described to be more concerned that 99DOTS use would expose them to TB stigma and to be more likely to have mobile phone–access issues than men with TB. By contrast, men with TB not only had access to mobile phones but also received substantial support from their female partners to take their anti-TB medication and make 99DOTS dosing confirmation calls. Finally, although women with TB were described to face more barriers to 99DOTS use than men with TB, the women’s narratives centered on the ways the platform facilitated and improved their adherence, whereas the men’s narratives did not.

**Conclusions:**

Overall, 99DOTS seems to be a feasible and acceptable strategy to support anti-TB medication adherence in Uganda. However, access to mobile phones, inability to charge mobile phones, and concerns about stigma should be considered and addressed as part of programmatic implementation to maximize uptake among all people with TB, particularly women and those with fewer financial resources.

## Introduction

### Background

Despite being preventable and curable, tuberculosis (TB) was the leading infectious disease cause of death worldwide before the COVID-19 pandemic [1]. Poor medication adherence remains a substantial obstacle to TB elimination [1]. Since the 1990s, directly observed therapy (DOT), wherein a health worker observes the swallowing of each dose of anti-TB medication, has been a key component of the standard of care for TB treatment supervision [2]. DOT is costly, it is time-consuming for people with TB and health workers, and there is limited evidence to suggest that it improves treatment outcomes [3]. Indeed, TB treatment success rates remain below the 90% target in most high-burden countries [1].

Digital adherence technologies (DATs) have recently emerged as an alternative to DOT. Common DAT platforms capture adherence using mobile phone–based SMS text messaging or calls, electronic pill boxes, or video-observed therapy [4]. DATs allow people with TB to take anti-TB medication at home, while generating real-time adherence data to enable health workers to monitor and support adherence remotely [5].

99DOTS (Everwell Health Solutions) is a low-cost DAT originally developed in India that involves people with TB calling toll-free numbers every time they take their anti-TB medication to report dosing. The toll-free numbers are ordered in an unpredictable pattern and hidden underneath pills in blister packs such that they are revealed only when patients remove a dose. Health workers can access adherence data for individual patients in real time through a web dashboard or mobile phone app. Automated SMS text messaging dosing reminders can also be sent daily to patients.

In the first randomized trial of 99DOTS conducted between December 1, 2018, and July 31, 2019, we found that 52% of the eligible people with TB at 18 health facilities in Uganda were enrolled in 99DOTS during the intervention period [6]. Treatment outcomes were similar during the intervention and control periods among all people with TB but were improved among those enrolled in 99DOTS. The largest improvements occurred in men and people living with HIV, although the subgroup differences were not statistically significant. These findings, as well as similar results from other studies of 99DOTS-based treatment supervision [7,8], highlight the need to better understand the factors that influence uptake and implementation outcomes.

### Objectives

To address the aforementioned need, we aimed to assess the feasibility and acceptability of 99DOTS among people with TB, health workers, and district and regional TB officers, as well as characterize barriers and facilitators to its implementation in Uganda.

## Methods

### Study Setting

We conducted semistructured in-depth interviews with people with TB using 99DOTS and key informant interviews with health workers and TB officers at 18 health facilities that were involved in our previous pragmatic trial of 99DOTS in Uganda [9]. The 18 health facilities included 5 regional referral hospitals, 10 district hospitals, and 3 county health centers and spanned across 15 districts within 225 km of Kampala, Uganda, as described previously [9]. The trial evaluated a version of 99DOTS that was adapted to the needs of people with TB and health workers in Uganda using human-centered design (HCD) methods [10,11]. Key changes included (1) adding a decorative cover to the pill pack to reduce stigma, (2) pictorial instructions for taking pills to address health literacy issues, (3) motivational images to promote adherence and health worker contact information to facilitate communication, and (4) replacing the ring tone heard by people with TB when making toll-free mobile phone calls to report dosing with a rotating series of educational and motivational messages recorded by health workers.

### Ethics Approval

The study received ethics approval from the Makerere University School of Public Health (approval 630), the University of California San Francisco (approval 18-26191), and the Uganda National Council for Science and Technology (HS 2518).

### Sampling and Recruitment

People with TB, health workers, and TB officers were recruited to participate in the study between April 1, 2021, and August 31, 2021. People with TB were eligible if they had been enrolled in 99DOTS at 1 of the 18 health facilities participating in the trial and started treatment during or after the trial (January 1, 2019, to May 31, 2021). People with TB were purposively sampled to ensure variability in age and health facilities, as well as equal representation by sex, HIV status, and engagement with 99DOTS (defined as high or low based on top vs bottom quartile, respectively, of call-in adherence).

Health workers were invited to participate if they served in the role of a clinical officer, nurse, or community health worker at 1 of the 18 health facilities and used 99DOTS in their role. We also recruited district and regional TB officers who were engaged in supporting 99DOTS implementation during the trial. Health workers and TB officers were purposively sampled to include those working at highly engaged versus minimally engaged health facilities (based on top vs bottom quartile, respectively, of proportion of patients enrolled in 99DOTS) as well as to ensure diversity in clinical role, facility location (rural vs urban), and facility type (health center vs hospital).

Trained study staff contacted selected participants by mobile phone to describe the study using a standardized recruitment script and schedule a separate time to participate in the interview if they agreed to participate. All participants provided verbal informed consent before starting interviews.

### Data Collection

Separate semistructured interview guides were developed for people with TB, health workers, and TB officers. The guides consisted of broad, open-ended questions meant to elicit participant perceptions of, and experiences with, 99DOTS. Interview guides were written in English and translated into Luganda. Questions in the guides were informed by prior research on these topics as well as the capability, opportunity, motivation, and behavior (COM-B) model [12], which posits that health behavior is shaped by 3 broad domains, including capability (eg, knowledge, memory, and attention), opportunity (eg, social influences, environmental context, and resources), and motivation (eg, optimism, reinforcement, and emotions) [12]. Accordingly, we included questions in the interview guides that aimed to explore each of the COM-B domains (Table 1).

**Table 1:** Capability, opportunity, motivation, and behavior (COM-B) domains explored in the semistructured interview guides.

COM-B domain			Example topics explored
**Capability**			
	Knowledge	Know how to use 99DOTS	
	Memory	Remember to use	
**Opportunity**			
	Social influences	TB^a^ stigma, support, and patient–health worker relationship	
	Environmental context and resources	Access to mobile phone, time commitment, and cost	
**Motivation**			
	Optimism	Perception of the impact of 99DOTS on TB treatment outcomes	
	Reinforcement	Reminders to use 99DOTS	
	Emotion	Fears of TB stigma	

^a^TB: tuberculosis.

Four trained, sex-matched interviewers from Uganda conducted the interviews. Interviews were conducted over the mobile phone in the participant’s preferred language (English or Luganda). If an interview was conducted over the mobile phone, interviewers began by asking the participant questions to confirm their identity and assess whether they were in a safe and private space to participate in the interview, following a standardized script. The interviews lasted between 45 and 60 minutes and were audio recorded, transcribed, and translated into English if necessary. All participants were assigned unique identification numbers and were compensated UGX 10,000 (approximately US $3) at the end of the interview for their time (following the Makerere University School of Public Health’s ethical guidelines for participant compensation).

During data collection, interviewers took detailed notes and met weekly with the rest of the study team to discuss the interviews they had conducted and the themes that emerged. Once thematic saturation was achieved (ie, no new themes emerged from the data), we stopped recruiting participants.

### Data Analysis

All interviews were entered into Dedoose software (SocioCultural Research Consultants, LLC) [13] for qualitative coding. Qualitative analysis was conducted using the framework approach to allow us to hone in on predefined areas of interest while also allowing new themes to emerge from the data [14]. Three researchers (AL, JG, and CB) developed an initial codebook consisting of a priori codes informed by the research questions, previous research on DAT implementation, and COM-B domains. The codebook was then iteratively refined as data analysis proceeded by adding new inductive codes to reflect emergent themes. This process continued with regular discussion until consensus formed around a final codebook. When the codebook was finalized, all codes were systematically applied across all transcripts. We began by double coding the transcripts with frequent meetings to discuss discrepancies in double coding and emergent themes until consensus was reached. We double coded until discrepancies were minimal such that approximately one-quarter of all transcripts were double coded. The remaining transcripts were single coded. The data were then organized into a chart format for each key theme that included summaries of different perspectives and experiences from several participants [14]. We compared and contrasted data across different themes and perspectives to facilitate the identification of associations between themes and potential explanations for key findings [14].

## Results

### Participant Characteristics

We conducted in-depth interviews with 30 people with TB (Table 2) and key informant interviews with 12 health workers (n=3, 25%, community health workers; n=7, 58%, nurses; and n=2, 17%, clinicians) and 7 TB officers (n=3, 43%, regional TB and leprosy supervisors and n=4, 57%, district TB and leprosy supervisors) engaged in 99DOTS at 18 health facilities in Uganda. Nearly half the health workers (5/12, 42%) interviewed were female, and half (6/12, 50%) worked in facilities that were minimally engaged in 99DOTS.

**Table 2:** Demographic characteristics of participants with TB (n=30).

Characteristics	Values
Age (years), median (range)	36 (22-70)
Sex (female), n (%)	16 (53)
Living with HIV, n (%)	17 (57)
Minimally engaged in 99DOTS, n (%)	16 (53)

### Dominant Themes

**Figure 1:**
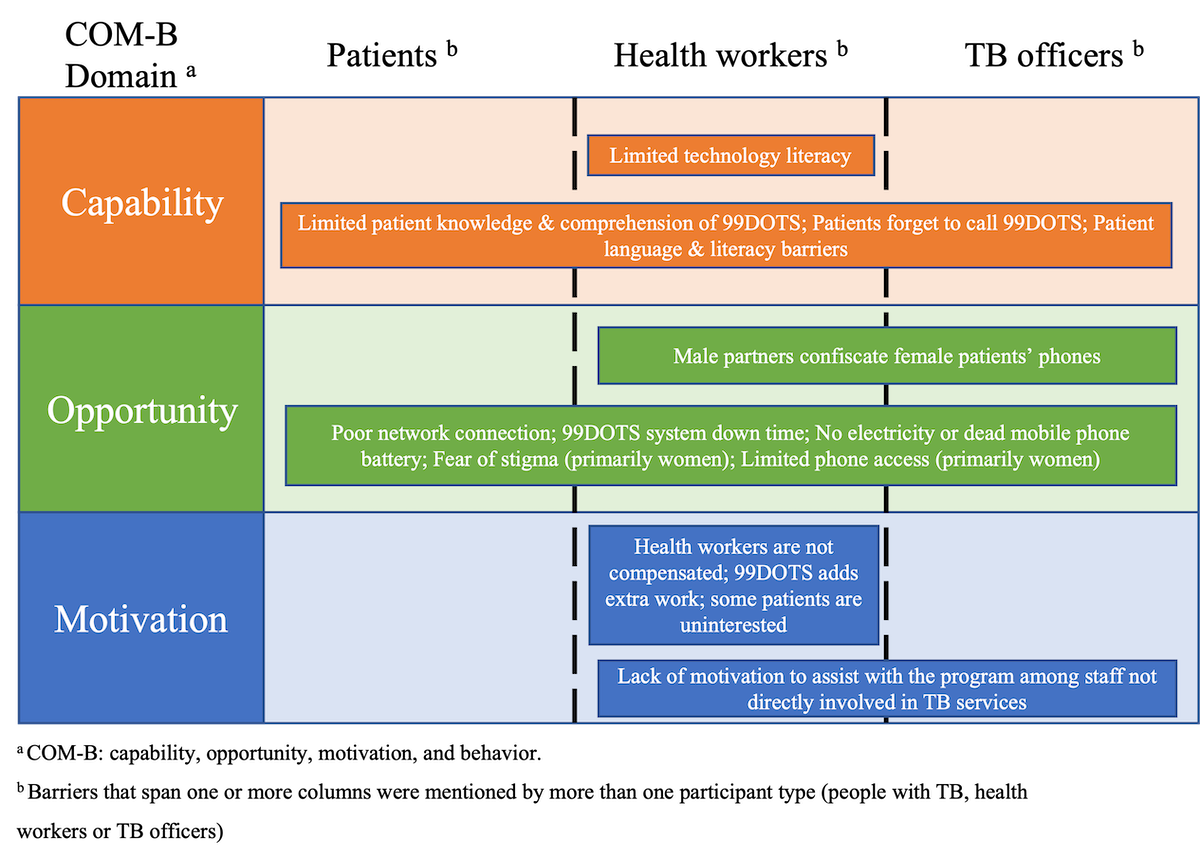
Barriers to the 99DOTS implementation in Uganda among people with tuberculosis (TB), health workers, and TB officers.

**Figure 2:**
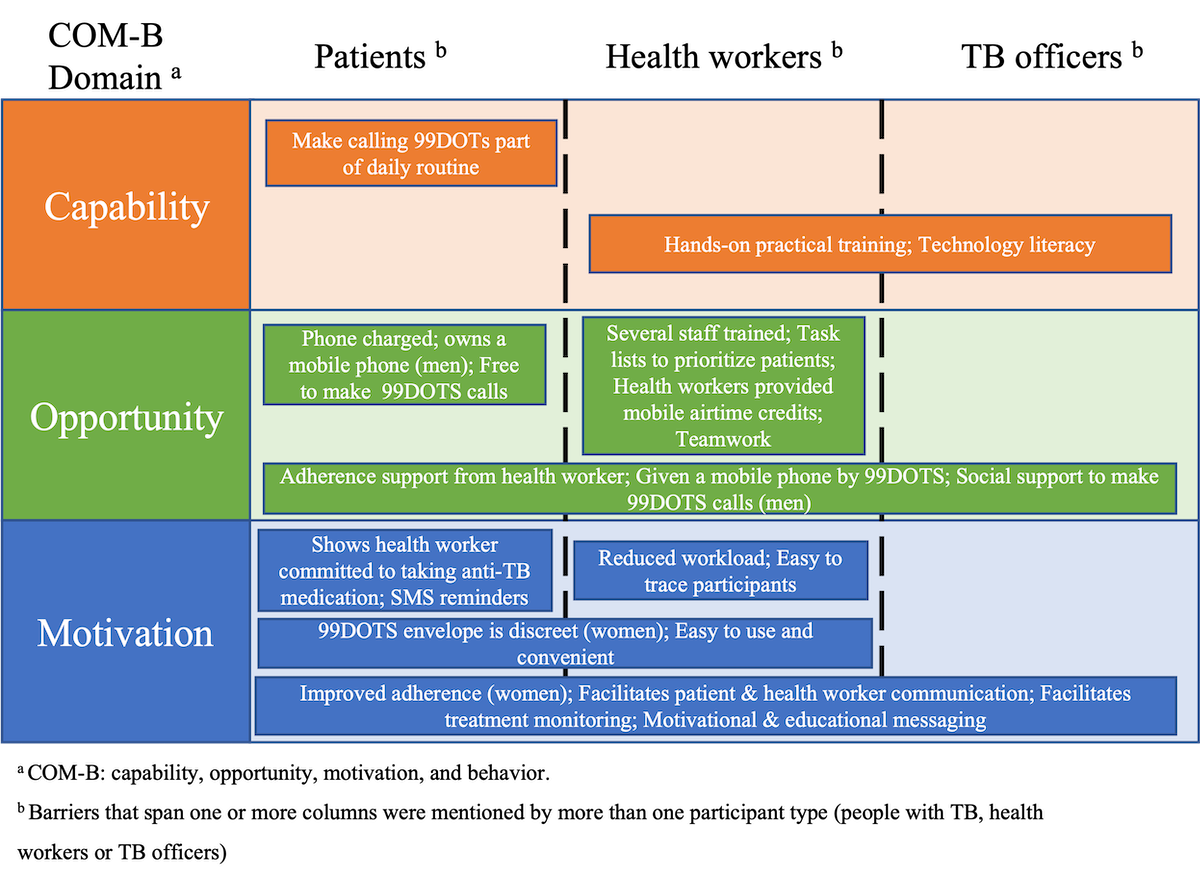
Facilitators of the 99DOTS implementation in Uganda among people with tuberculosis (TB), health workers, and TB officers.

The dominant themes that emerged from the 3 data sources mapped onto the 3 domains of the COM-B model: capability, opportunity, and motivation. Although the narratives from people with TB, health workers, and TB officers shared some common themes, we also identified unique themes by respondent type and sex. We did not find any differences by HIV status. We outline the dominant themes related to the barriers (Figure 1) and facilitators (Figure 2) to implementation of 99DOTS in Uganda, organized by COM-B domain.

### Capability: Comprehension and Remembering to Call 99DOTS

Some of the people with TB noted that they had difficulty remembering to call 99DOTS after taking their anti-TB medication daily and had limited knowledge or understanding of how to use 99DOTS. Health workers and TB officers also described these phenomena. Some of these participants explained that minimal comprehension of the system among some people with TB was driven by low literacy levels and language barriers. Specifically, the motivational audio messages and SMS text messaging reminders were all in Luganda, English, or Runyakitara. This posed challenges for people with TB who could not read or did not speak these languages.

Health workers who participated in our study also noted that limited technology literacy among some health workers and older people with TB was a barrier to 99DOTS implementation. Limited technology literacy was not raised as a barrier to 99DOTS by people with TB themselves. However, health workers noted that people with TB who had limited technology literacy said that they would try to get a neighbor or a child to help them use the mobile phone, but this approach had its flaws, for example, support people were not always available to help people with TB call 99DOTS when they took their medicine, leading them to miss calling 99DOTS after dosing and being incorrectly classified as having not taken their medication. A nurse described this phenomenon in the following way:

Some patients are too old...but if they have someone to help them with the phone, they can really help. Though you may find someone who is helping that patient is not there to help them with the phone all the time.

Female nurse, rural hospital highly engaged in 99DOTS

In terms of facilitators, several people with TB described incorporating taking their anti-TB medication and calling 99DOTS into their daily routine to help them remember to call the platform. Most health workers and TB officers described how hands-on practical training in the use of 99DOTS facilitated their understanding of the intervention and ultimately enabled them to implement the intervention successfully, with a TB officer commenting as follows:

We were given time to learn the system itself...We had all the time and we were allowed to ask questions and it was actually not hard because we were given phones and not taught on the blackboard, we were given phones to practice what we were being taught.

Male district TB supervisor

Several health workers and TB officers also noted how health workers with existing technology literacy found it easier to learn the program quickly.

### Opportunity: Stigma, Social Support, Access to Resources, and Gender-Based Differences

Members from all participant groups identified poor network connection, limited access to electricity, and technical issues with the 99DOTS platform, such as the system being down, as barriers to using 99DOTS. More female participants than male participants with TB stated that they had unreliable access to a mobile phone. Some of the female participants said that their mobile phone had been stolen, whereas others said that they shared a mobile phone with a family member because they did not own one themselves. A dominant theme that emerged from health worker and TB officer interviews offered some additional insights into why women were more likely to have limited access to mobile phones than men: some male partners confiscated the 99DOTS phones given to women with TB owing to the belief that the mobile phones would facilitate romantic relationships with other men. A nurse commented on this phenomenon as follows:

When it comes to women, men don’t want their wives to be given phones. They think they will be seduced on the phone. So before giving them the phone, we first tell the husband that this phone is for the hospital. She’s going to be with it for six months and it is going to just help her take her medicine. But still...I gave [a mobile phone] to a lady and the husband still removed the phone from her and she could not call.

Female nurse, rural hospital highly engaged in 99DOTS

However, this phenomenon was not described by either female or male participants with TB.

To overcome the barrier of limited mobile phone access, some of the female participants said that they borrowed a mobile phone from a family member, friend, or neighbor to call 99DOTS. However, access to borrowed mobile phones was not always reliable. In the following quote, a female participant who borrowed her sister’s mobile phone to call 99DOTS describes her challenges calling the system in a timely manner:

A person without a phone can’t use this method well [if] they are borrowing a phone...In case [my sister] had moved out [left the house] and I had to call, as soon as she came back I would tell her I took the medicine so I needed to call and we would call...I would take my medicine on time but only because the owner of the phone was not around I would call later once she got home.

Female participant, aged 39 years, HIV negative, minimally engaged in 99DOTS

Another strategy some of the female participants used to call 99DOTS when they lacked access to their own mobile phone was to call the system using mobile phones that were not registered in the system. However, the system does not recognize calls from unregistered numbers and would therefore incorrectly classify some people with TB as having missed their dose for a particular day although a call was made.

The fear of experiencing TB stigma also emerged as an important barrier to 99DOTS use, more so for female participants with TB than for male participants with TB. A few of the female participants described how their husbands left them after learning of their TB status owing to the misperception that someone with TB must also be living with HIV, as described in the following quote:

He didn’t believe [that I am HIV negative]. He said that I have HIV. So, he got another wife. He went and checked his HIV status, and he was told he was negative so he said he couldn’t come back to me who was HIV positive.

Female participant, aged 27 years, HIV negative, highly engaged in 99DOTS

Several of the female participants described keeping their TB status a secret owing to their fear of being abandoned by their husbands and experiencing TB stigma from their community. Some of the female participants asserted that these fears made them hesitant to use 99DOTS because they worried that their use of the platform would lead to accidental disclosure of their TB status and ultimately expose them to TB stigma. This concern was raised particularly in relation to the SMS text messaging reminders, which the female participants feared others would see on their mobile phone, as explained by a female participant:

Another bad thing I faced is when someone else was in my presence and the phone rang. That thing scared me...The doctors would call to check if I was taking the medicine...And also the [SMS text] messages were risky because someone might touch your phone and read them.

Female participant, aged 31 years, HIV positive, minimally engaged in 99DOTS

The finding that female participants’ concerns about TB stigma prevented their engagement in 99DOTS was supported by health worker and TB officer narratives:

Some women...they don’t want to be known that they have TB. The issue of stigma is more in women than men. So...if for example she [uses the] phone of her husband, she would rather not use that phone for alerts for 99DOTS.

Male district TB supervisor

In terms of facilitators, most of the people with TB described how being able to keep their mobile phone charged and the fact that it was toll-free to make 99DOTS calls facilitated their engagement. Some of the health workers described how having several staff trained to use 99DOTS improved the implementation of the intervention because the implementation did not rest on a select few health workers—there were enough people trained to keep the program running even when a health worker was out of the office. The health workers also described how the extent to which a group of health workers were able to work as a team improved implementation success, for example, health workers described how those who were more confident using technology would train and support health workers who were not as technology savvy. This helped to ensure that all team members could participate in the implementation. Health workers also liked how the 99DOTS platform included a task list to prioritize patients in need of extra counseling and attention.

Finally, male participants with TB were more likely than female participants with TB to note that the social support they received to make 99DOTS calls (mainly from their female partners) helped them to make dosing confirmation calls, as described in the following quote (this was supported by health workers and TB officers):

I tried my level best [to remember to call 99DOTS]. My wife was there to remind me. If I didn’t call, she would tell me to call. It’s like we were two patients. As if she was also suffering. That’s how it was.

Male participant, aged 34 years, HIV positive, highly engaged in 99DOTS

### Motivation: Adherence Reinforcement, Impact on Health Worker Workload, and Acceptability

Barriers to 99DOTS implementation that related to the motivation domain of the COM-B model primarily emerged from health worker and TB officer narratives. A few of the health workers noted that some of their colleagues disliked that they were not compensated for their participation in 99DOTS and therefore did not contribute to the intervention in the way that they should have. However, this was not a dominant theme. A few of the health workers also noted that some of their colleagues felt that 99DOTS just added extra work, such as checking on adherence and sending adherence reminders after work hours, which prevented their engagement in the intervention.

In terms of facilitators, most of the health workers asserted that 99DOTS reduced their workload because it improved their ability to trace people with TB and helped them to monitor and support adherence to anti-TB medication. People with TB, health workers, and TB officers all asserted that the platform was convenient and easy to use. Health workers and TB officers liked how 99DOTS allowed them to monitor adherence remotely:

[99DOTS] usually eases the work. You move with your patients at hand, on the phone...At any time even if you are away from the facility, you can check...You have access to the information wherever you are.

Male nurse, rural hospital minimally engaged in 99DOTS

Most of the people with TB liked that they were able to take their medication at home and did not have to travel to the hospital for DOT, which saved time and transport costs.

Female participants with TB were more likely than male participants with TB to state that they liked that the pill pack envelope was discreet so that no one could tell that they were taking anti-TB medication. Health workers also mentioned this preference among female participants with TB. In the following quote, a female participants with TB describes how the discreet design of the pill pack envelope helped her adhere to her anti-TB medication:

[I]n case you are traveling with your packet you don’t feel scared to pull it around people. Not even anyone will get to know that you’re swallowing [TB] medicine because [of] the way you made it...The person just sees tablets because they are covered, you just open a little bit and remove some, then cover it back...they are better than the other sachets where you get a sachet, and someone gets scared at what you are swallowing. Here you just remove it and, on the cover, it has flowers. Someone doesn’t get to know what you are getting...you can swallow from the taxi or you can even swallow around people, let me say those who [might] discriminate you.

Female participant, aged 24 years, HIV negative, highly engaged in 99DOTS

People with TB, health workers, and TB officers all asserted that they liked how 99DOTS enabled health workers to monitor patient adherence in real time and offer adherence counseling and support. Most of the people with TB described how knowing that health workers could see whether they took their medication, as well as the encouragement and support they received from health workers through their calls and automated messages, motivated them to take their medication:

[99DOTS] helps gain the doctor’s trust in us that, this one swallows every day. It also helps you not to cheat, that let me miss today and make a phone call that I swallowed. It bothers you at heart that you didn’t swallow the medicine so you can’t make a phone call.

Female participant, aged 24 years, HIV negative, highly engaged in 99DOTS

The [health worker] phone calls helped me to be strong and I felt well about it. It’s like when you are feeling discouraged and feeling like dying, and someone calls you and encourages you.

Female participant, aged 37 years, HIV positive, minimally engaged in 99DOTS

People with TB and health workers both asserted that 99DOTS established a line of communication between them and allowed health workers to offer real-time adherence support, something that all participants noted did not exist before. A health worker described this phenomenon in the following way:

With 99DOTS, it has been useful to me in that I know how my patient is doing. I can monitor the adherence of my patient to [TB] drugs. I can communicate to my patient at any time. There’s that bridge between me and my patient. Yes, so the patient is very free to talk to me where he finds a challenge and I’m able to address that challenge, if possible, on the phone. If it needs the patient to come to the facility, then I’ll tell the patient to come to the facility. Yes, [before 99DOTS] if the patient faced a challenge, it meant the patient will suffer with the challenge until he comes to the facility to find a health worker, which isn’t good.

Female nurse, urban hospital minimally engaged in 99DOTS

Real-time adherence monitoring and support was described by participants in all groups to strengthen the relationship between people with TB and health workers. In addition to people with TB “gaining the doctor’s trust,” as described by the female participant with TB previously, people with TB also noted that health workers contacting them to offer encouragement and support strengthened their own trust in health workers and their belief that health workers truly cared about their well-being:

[99DOTS] helped me to know that [health workers] take the initiative to find out that the patients are taking their medicine because you can find doctors who don’t care but it showed me that they are always thinking about the patients and have a responsibility to call to find out how the patients are and whether they are taking their medicines or not.

Female participant, aged 46 years, HIV positive, highly engaged in 99DOTS

Health workers, TB officers, and female participants with TB (more than male participants with TB) reported that these aspects of 99DOTS, which facilitated real-time adherence monitoring and support, played a critical role in improving treatment outcomes. A TB officer described the positive impact 99DOTS had on treatment outcomes for facilities in his district:

[99DOTS] has improved adherence to treatment because [patients] are given reminders. They are given phone calls...Then the health worker–patient relationship is good...[patients] are in touch with the health worker almost all the time. Then the treatment success rate of course with the adherence and patients taking their medicines...the success rate has improved.

Male district TB supervisor

In the following quote, a health worker offers additional insight into the ways 99DOTS improved treatment outcomes in their clinic:

I was so grateful to the system...before 99DOTS...it was too tricky to follow up, trace patients, and ensure proper adherence for these patients...It became quite difficult to follow up these patients especially during the continuous phase...these patients could no longer adhere to their medication or honor their appointments. But at least the 99DOTS...it closed the gap between you the health worker and your patient. In other words, the patient could still feel that you really do have that much care for them...we no longer have so many lost to follow up, our patients take their drugs very well and they complete...our cure rate has actually increased, even completion increased, [and] it was due to this system.

Female community health worker, rural hospital with minimally engaged in 99DOTS

### Recommended Changes to 99DOTS

When asked what they would change about 99DOTS, people with TB emphasized the importance of the program providing mobile phones to those who did not have them. In addition, female participants with TB, in particular, articulated the need for the SMS text messages to be more discreet to address concerns of TB stigma. Both people with TB and TB officers emphasized the importance of offering the intervention in several languages to ensure that as many people with TB could benefit as possible.

Health workers identified several recommended changes to the 99DOTS dashboard to improve their reporting and participant tracking; for example, health workers recommended the addition of a data-capture feature for HIV status to the dashboard so that they can easily remember which patients were living with HIV and tailor their counseling accordingly. Both health workers and TB officers suggested that 99DOTS be scaled up to other clinics. Health workers also suggested that changes be made to the health worker training, including training more clinic staff on how to use 99DOTS and to conduct off-site trainings to limit distractions from their clinical duties during the training. Most of the health workers also suggested that 99DOTS also be offered to children and adolescents because they also face adherence challenges:

Another thing that we should think of is also adding or enrolling children...you [should] develop that system also for children because we have noticed that these young children get lost and their mothers or caretakers ignore it or sometimes they realize that this child has improved a bit and they don’t turn up or they don’t come back...you people [should] think of bringing children on board, such that they are also able to be monitored well.

Male nurse, rural hospital with minimally engaged in 99DOTS

To address the barriers that women with TB faced in engaging with 99DOTS, particularly related to concerns about TB stigma and male partners confiscating their mobile phones, TB officers suggested that male partners be included in the initiation of 99DOTS for female patients. They suggested that male partners not only be sensitized to why female patients are being given a mobile phone to monitor their treatment adherence but also be sensitized to the fact that TB is curable and not necessarily a sign that someone is living with HIV:

[I]f it’s a woman who has come, arrangements to give her a phone could be made when the woman is hearing and the husband is hearing too so that they know the importance of the phone, the importance of calling daily so that there are no wrangles. So, make [it] known to the family why you are giving them the phone, so that the [husband] knows that the phone is not because the health worker has admired [his wife] but it’s for the whole family to help comply with treatment.

Male district TB supervisor

Finally, people with TB and TB officers also highlighted the need for structural interventions to augment the 99DOTS intervention to address the social and structural barriers to engagement in 99DOTS and adherence to anti-TB medication. Specifically, TB officers identified the need for broader efforts to combat TB stigma in the community to facilitate women’s adherence to TB treatment and engagement in 99DOTS:

I think we need a general sensitization over radio, on televisions...you hear people talking of TB over radio, they say, “If you have TB, you might be having HIV,” so even that one I think has caused problems because if you are a man and you hear your wife has TB and you hear that over the radio, then you want to do some other investigation to find out whether this woman also has HIV. But definitely we need to do more of the sensitization through media such that it is known that you can have TB and TB is curable.

Male district TB supervisor

People with TB also noted the need for 99DOTS to be offered in tandem with food support. Several people with TB identified limited food access as a main barrier to anti-TB medication adherence, either owing to the belief that anti-TB medication cannot be taken on an empty stomach or because of the need to use their limited money on food for their family instead of traveling to the clinic to collect their anti-TB medication. Several of the people with TB suggested that if they had reliable access to food, they would be better able to adhere to their medication:

If the doctor gives you medicine and you don’t have what to eat or drink you don’t get better fast...because you have to look for something to eat by yourself, you might even have TB for a whole year without getting cured...but if there is a service at the hospital that the TB patients, we should give them some soya or porridge, it quickens it for the patient to reduce on virus and heals very fast. So that the patient does not spread it to many others.

Female participant, aged 30 years, HIV negative, minimally engaged in 99DOTS

## Discussion

### Key Findings and Their Implications

Our findings suggest that, in general, 99DOTS is highly acceptable among people with TB, health workers, and TB officers in Uganda. Specifically, participants across all groups liked how 99DOTS facilitated real-time treatment monitoring and support, increased convenience of accessing TB treatment, and improved connections among patients and health workers. However, unreliable access to mobile phones, inadequate social support, and concerns about stigma emerged as critical barriers to 99DOTS uptake and engagement, particularly among female participants with TB as well as those with TB who have fewer financial resources. In addition, broader efforts to combat stigma and address socioeconomic barriers, including food insecurity, are needed to improve TB treatment outcomes.

Although past studies examining the implementation of 99DOTS and other DATs have documented concerns about reduced communication between people with TB and health workers [7,15], participants in our study perceived 99DOTS to improve connection between them. This was described to be facilitated by (1) health workers calling people with TB who had not made their dosing confirmation calls; (2) the inclusion of the health worker’s mobile phone number on the 99DOTS pill pack; and (3) the educational and motivational messages that patients heard from health workers when they made 99DOTS dosing confirmation calls. Importantly, features 2 and 3 were adaptations our group made to the 99DOTS platform based on our HCD work [10,11]. Our prior HCD work also informed the adaptation of the 99DOTS pill pack to make it more discreet with the goal of reducing stigma. The findings from this study revealed that the adapted discreet pill pack facilitated anti-TB medication adherence among women with TB in particular. Taken together, these results highlight the utility of using an HCD process to inform the design and adaptation of DATs to better meet the needs of end users.

Our findings also revealed important gender-based barriers to 99DOTS use. Specifically, we found that women with TB lacked reliable access to mobile phones and reported fears of experiencing TB stigma, which impeded their engagement in 99DOTS. Health worker narratives suggested that some male partners went so far as to confiscate the mobile phones given to women with TB. By contrast, most of the male participants with TB reported owning their own mobile phone and described receiving social support to make calls, primarily from their female partners. These findings suggest that inequitable gender norms may have shaped the implementation of 99DOTS in this setting. Inequitable gender norms can condone men’s dominance over household decision-making and control over women [16,17]. They can also include norms around men’s role as the primary provider and women’s role as the caregiver [18]. These gender norms are prevalent in Uganda and other parts of sub-Saharan Africa [19,20] and drive gender-based economic disparities [21]. These norms likely shaped women’s unreliable mobile phone access and the support men with TB received from their female partners to complete 99DOTS dosing confirmation calls, which reflect gender norms emphasizing women’s primary role as family caretaker [18]. Our finding that TB stigma was a key barrier to 99DOTS uptake among women with TB can also be understood through the lens of inequitable gender norms. The results from this study and others [22-24] suggest that TB stigma is linked to assumptions about HIV status and that women with TB are viewed as promiscuous and are often rejected by their partners, with potentially severe economic and social impacts.

Finally, although women with TB faced more barriers to engaging in 99DOTS than men with TB, the women’s narratives centered on the ways the platform itself facilitated and improved their adherence when they did use it, whereas the men’s narratives did not. It is possible that the adherence support offered through the 99DOTS platform was particularly useful for the women who were able to access the system, especially in the absence of other forms of social support. The fact that the men’s narratives did not focus on the adherence support offered by 99DOTS may be because many of the men with TB described access to existing adherence support from their female partners. Health workers and TB officers also expressed their perception that 99DOTS improved TB treatment outcomes.

Study limitations included only interviewing people with TB who were engaged in 99DOTS; therefore, we did not capture the barriers to implementation among those not enrolled in 99DOTS. However, we structured our sampling frame to ensure equal representation of people with TB with low and high engagement in 99DOTS to gain as comprehensive an understanding of the barriers and facilitators to implementation as possible. It is also possible that participants may not have felt comfortable providing critical feedback in relation to the intervention. To mitigate this, we used experienced interviewers trained in qualitative methods, including neutral probing and strategies to establish rapport, to help participants feel comfortable sharing their honest perspectives of the intervention. Finally, the fact that we recruited participants by mobile phone may have limited our ability to recruit people with TB who had limited access to a mobile phone and who may have experienced additional barriers to participating in 99DOTS.

### Conclusions

In summary, 99DOTS seems to be an acceptable and feasible strategy to support anti-TB medication adherence in Uganda. The findings highlight the importance of adapting DATs to better meet the needs of people with TB and indicate the need for more material and social support services, particularly for women accessing mobile phone–based DATs. The results from this study suggest that when women with TB have the social and material means to engage in 99DOTS, they greatly benefit.

The results from this study also suggest the need for programmatic efforts to address TB stigma and inequitable gender norms related to women’s mobile phone use; for example, male partners of women with TB could be invited to attend TB appointments and educated about TB and foster male partner support for TB treatment adherence and engagement in 99DOTS, as suggested by TB officers in this study. Other research from sub-Saharan Africa has demonstrated that couples-based health education and adherence counseling is a successful strategy to improve treatment adherence for HIV [25,26]. In addition, providing low-cost mobile phones to people who need them or DATs that do not require frequent charging or consistent network connections may be needed to maximize uptake.

## Abbreviations

COM-B: capability, opportunity, motivation, and behavior
DAT: digital adherence technology
DOT: directly observed therapy
HCD: human-centered design
TB: tuberculosis
